# Effects of the supernatant of *Chlorella vulgaris* cultivated under different culture modes on lettuce (*Lactuca sativa* L.) growth

**DOI:** 10.3389/fnut.2024.1437374

**Published:** 2024-08-30

**Authors:** Lin Dai, Peng Yu, Pengyao Ma, Cheng Chen, Jun Ma, Jinli Zhang, Bo Huang, Zhikun Xin, Xufan Zheng, Tao Tang

**Affiliations:** ^1^ChnEnergy XinJiang TuoKexun Energy Co., Ltd., Xinjiang, China; ^2^School of Civil and Resources Engineering, Graduate School of University of Science & Technology Beijing, Beijing, China; ^3^ChnEnergy New Energy Technology Research Institute Co., Ltd., Beijing, China; ^4^CAS Key Lab of Low-Carbon Conversion Science & Engineering, Shanghai Advanced Research Institute, Chinese Academy of Sciences, Shanghai, China; ^5^State Key Laboratory of Low Carbon Catalysis and Carbon Dioxide Utilization, Shanghai Advanced Research Institute, Chinese Academy of Sciences, Shanghai, China

**Keywords:** *Chlorella vulgaris*, cultivation modes, supernatant, lettuce, fertilizer

## Abstract

CO_2_ capture by microalgae is a feasible strategy to reduce CO_2_ emissions. However, large amounts of cell-free supernatant will be produced after microalgal harvesting, which may be harmful to the environment if it is disorderly discharged. In this study, *Chlorella vulgaris* (*C. vulgaris*) was cultivated under three common cultivation modes (autotrophic culture (AC), heterotrophic culture (HC) and mixotrophic culture (MC)), and the obtained supernatant was used as fertilizer to investigate its effect on the growth of lettuce. The biomass concentration of *C. vulgaris* cultivated under MC and HC was 3.25 and 2.59 times that of under AC, respectively. The contents of macronutrients in supernatant obtained from AC were higher than those of MC and HC. However, the contents of amino acids and hormones in supernatant obtained from MC and HC were higher than those of AC. The fresh shoot weight, fresh root weight and root length of lettuce treated with supernatant were significantly higher than that of control treatment. In addition, the contents of chlorophyll, soluble sugar and soluble protein in lettuce treated with supernatant were also higher than that of control treatment. However, the contents of nitrate in lettuce treated with supernatant was lower than that of control treatment. These results showed that the supernatant could promote the growth of lettuce and was a potential of fertilizer for crop planting.

## 1 Introduction

Atmospheric CO_2_ concentration has risen by 95 ppm over the last 100 years since the industrial revolution. CO_2_ is the largest contributor to the greenhouse effect, and the reduction of CO_2_ level will directly affect the total greenhouse gas emissions. Although there are different CO_2_ capture approaches, the biological CO_2_ capture method is a potentially attractive alternative. The rapid growth rate of microalgal cells provides a competitive advantage in carbon sequestration. The carbon sequestration efficiency of microalgae is 10–50 times that of terrestrial plants (1, 2). Moreover, microalgal biomass contains high amounts of primary metabolites and secondary metabolites. These metabolites are useful feedstock for food, feed, energy and high value products. Therefore, microalgal cultivation emerges as an attractive alternative to carbon capture.

A lot of water and nutrients are required in the process of microalgal cultivation, and then a lot of cell-free supernatant will be produced after microalgal harvesting. The supernatant will be harmful to the environment if it is disorderly discharged. Generally, recycling/recovery/reuse of cell-free supernatant is a common method used in traditional microalgal cultivation as it can lead to massive saving in water and chemical consumption, which is essential for the economic viability and sustainability of microalgal cultivation (3). One life cycle analysis for microalgal biodiesel production determined that reusing water could reduce freshwater demand by 84% and nutrient usage by 55% (4). However, organic matter accumulation in the cell-free supernatant, such as polysaccharides, proteins, free fatty acids and cell debris (5–7), which may likely to promote bacterial growth and decrease microalgal growth. In addition, the recycled medium can also affect the cellular lipid, pigment, carbohydrate and protein contents in microalgal cells (5, 8–12). Recently, pretreatment methods have been developed to remove the growth inhibitors in cell-free supernatant. Granular activated carbon absorption, high pH flocculation and ferric chloride flocculation were effective in removing a substantial portion of organic matter in the reused water (13–15). Other studies have shown UV-based advanced oxidation processes UV/peroxydisulfate and UV/H_2_O_2_ are a promising pre-treatment technique (16, 17). Thus, while water reuse is generally recognized as desirable, barriers exist to broad implementation.

Microalgal extracts have been widely shown to be biostimulants for higher plants (18–26). *Ulothrix* sp. and *Klebsormidium* sp. were used as a high-value organic slow-released bio-stimulant for tomatoes cultivation, which resulted in the increase of the contents of carotenoid and sugar levels (19). Puglisi et al. (24) investigated the effect of the microalgal extracts from *Chlorella vulgaris* or *Scenedesmus quadricauda* on the sugar beet germination. It was found that these microalgal extracts exerted a positive effect on sugar beet germination. Mieczyslaw et al. (23) found that polysaccharides of *Cyanobacteria* and *Chlorella* species can be beneficial to the growth, development, seed germination, and metabolic activity of corn. Gitau et al. (20) systematically investigated the plant-growth-promoting effects of *Chlorella* isolates (MACC-360 and MACC-38) and *Chlamydomonas reinhardtii* (cc124). It was found that *Chlorella* application led to more robust plants with increased fresh biomass, bigger leaves and more flowers/pods compared to the control and *Chlamydomonas*-treated samples receiving identical total nutrients. Microalgal extracts possess many growth-promoting properties which include carbohydrates, proteins, lipids, vitamins, micronutrients, macronutrients, and phytohormones. These metabolites are also secreted into the culture medium in the process of microalgal cultivation. In addition, large amounts of residual inorganic salts remain in the culture medium. Therefore, the supernatant may be used as a kind of fertilizer for crop planting.

In this study, we investigated the feasibility of microalgal supernatant as fertilizer for crop planting. *C. vulgaris* was cultivated under three common cultivation modes, and the obtained supernatant was used as fertilizer to investigate its effect on the growth of lettuce.

## 2 Materials and methods

### 2.1 The cultivation of *Chlorella vulgaris* and lettuce

*Chlorella vulgaris* was separated from a water sample collected locally (Shanghai), and maintained in petri dishes using BG11 solid medium. *C. vulgaris* cells were successively transferred from petri dishes to 250 mL flasks, and then cultivated in 400 mL bubble column photobioreactors with 1% CO_2_ under 100 μmol m^−2^ s^−1^ and 25°C conditions. The cells of *C. vulgaris* were harvested during their logarithmic growth phase by centrifugation. Discarded the cell-free supernatant and rinsed the harvested cells surface with sterile distilled water to remove bacteria. All the harvested cells were resuspended into the required culture medium and used in the following experiments.

Three common cultivation modes were selected for *C. vulgaris* cultivation, including autotrophic culture (AC), heterotrophic culture (HC) and mixotrophic culture (MC), with three replicates for each mode. For AC, *C. vulgaris* was cultured in a 1 L Erlenmeyer flask (working volume 800 mL) with an initial concentration at OD_680_ of 0.05, and the culture conditions were as follows: the culture medium was BG11 medium, CO_2_ concentration was 1% with air filtering by 0.22 μm filter membrane, the light intensity was 100 μmol m^−2^ s^−1^ and the room temperature was 25°C. For HC, the culture medium was BG11 medium, in which 20 g/L glucose was added as the only carbon source and energy source. The light and CO_2_ conditions were removed. The air filtered by the filter membrane (0.22 μm) was used for uniform mixing of microalgal cells. For MC, 20 g/L glucose were added into BG11 medium as the culture medium, and other cultivation conditions are the same as AC. All the treatments were static in bench shelves with the aeration rate of 0.3 L/min. Samples were taken every day to measure the optical density and dry weight of *C. vulgaris* cells. The cells of *C. vulgaris* were harvested after 4 days in MC and 5 days in AC and HC. The culture was centrifuged at 6000 rpm for 10 min to collect the cell-free supernatant, and the obtained cell-free supernatant was stored at −20°C until use.

Seeds of lettuce (*Lactuca sativa* L.) cultivar “Grand Rapids” (USA) were soaked in deionized water at 30°C for 4 h to improve germination rate. The pre-mixed nutrient soils (organic fertilizer and coconut bran, 1:1 (v/v)) were put into the seedling trays and watered thoroughly. The seedling trays were cultured in an artificial climate box. The temperature was set at 25°C during the day and 20°C at night. After germination, keep two lettuce seedlings in each hole. Seedlings with 3–4 true leaves were transplanted.

### 2.2 Lettuce growth experiment

In order to investigate the effect of supernatant of *C. vulgaris* cultivated under different cultivation modes on the growth of lettuce, four treatments were set up, which included treatment 1 (control, CK), treatment 2 (mixotrophic culture, MC), treatment 3 (heterotrophic culture, HC) and treatment 4 (autotrophic culture, AC) with six replicates for each treatment. Each pot contained 2 L of nutrient soils (organic fertilizer and coconut bran, 1:1 (v/v)). 24 lettuce plants with the same size and growth state were selected and one seedling of the lettuce was grown in each pot. Fertilizer treatment was carried out on the day of transplanting lettuce. Every pot was firstly irrigated with 100 mL of garden nutrient solution (GNS), and then 50 mL supernatant obtained from MC, HC and AC were correspondingly added to treatment 2, 3, and 4, respectively. At day 4, 50 mL of water was added to the control group. 50 mL of the obtained supernatant was added to AC, HC and MC groups, respectively. In addition, all the treatment groups were irrigated with 100 mL of GNS at day 7 and day 11, respectively. Table 1 shows the amounts of fertilizer applied in each treatment group.

**Table 1:** The amounts of fertilizer applied in each treatment group.

Treatment	0d	4d	7d	11d
T1 (Control)	50 mL water + 100 mL GNS	50 mL water++100 mL GNS	100 mL GNS	100 mL GNS
T2 (HC)	50 mL supernatant + 100 mL GNS	50 mL supernatant + 100 mL GNS	100 mL GNS	100 mL GNS
T3 (MC)	50 mL supernatant + 100 mL GNS	50 mL supernatant + 100 mL GNS	100 mL GNS	100 mL GNS
T4 (AC)	50 mL supernatant + 100 mL GNS	50 mL supernatant + 100 mL GNS	100 mL GNS	100 mL GNS

### 2.3 Analytical methods

#### 2.3.1 Optical density and dry weight of *Chlorella vulgaris*

5 mL sample was filtered using a pre-dried and pre-weighed cellulose membrane (0.45 μm pore size), washed with deionized water, dried for 24 h at 105°C, cooled in a desiccator and then weighed again to determine uncorrected dry algae biomass. The dry weight of the blank filter was subtracted from that of the loaded filter to obtain the corrected algae dry cell weight. The optical density of cells in culture was determined by the measured absorbance with an HACH-DR2800 spectrophotometer at 680 nm.

#### 2.3.2 Determination of the composition of supernatant

The composition of supernatant was examined using ICP-MS method, which contained total phosphorus (TP), total potassium (TK), calcium, magnesium, iron, copper, molybdenum (27). Weighed the appropriate amount of sample into the PTFE digestion tank and added 5 mL of nitric acid. After the reaction was finished, sealed the lid and put it into the microwave disintegrator. After the temperature was cooled down to below 50°C, took out the digestion tank and put it into a fume hood, opened the digestion tank, rinsed it with ultrapure water, transferred it to a 25 mL volumetric flask, rinsed it at least 3–4 times, diluted it with ultrapure water and set the volume to the scale, and then wait for the test. The blank control was treated in the same way. The total carbon (TC) and total nitrogen (TN) were analyzed by Total Organic Carbon Analyzer (Shimadzu, TOC-L, SSM-5000A, Japan; (28, 29)). The hormone components including auxin, gibberellin and abscisic acid were analyzed using LC–MS method (21). The amino acid contents were analyzed using HPLC method (30).

#### 2.3.3 Lettuce harvest and determination

The lettuce was harvested and sampled 15 days after transplanting. Three leaves per pot were selected and dark-adapted for 30 min using dark-adapted leaf clips to hold the lettuce leaves in place. The chlorophyll fluorescence quantum yield (*F*_v_/*F*_m_) was determined by Hansatech Fluorescence Monitoring System (FMS-2). The excess soil of lettuce roots was washed away and the residual water on the surface of lettuce was absorbed with absorbent paper. Then the root length, the fresh weight of shoot and root were measured, respectively. Representative lettuce samples were taken, chopped and mixed, and prepared as a homogenate using a tissue masher. Chlorophyll content was determined by ethanol-acetone extraction method according to the agricultural industry standard of China (NY-T 3082-2017). The soluble sugar was determined by Anthrone-colorimetric method (31), the vitamin C content was analyzed according to the national standard of China (GB 5009.86-2016) and the vitamin E content was measured by kit (Beijing Solarbio), the soluble protein was determined by Coomassie brilliant blue-staining method (31), and the nitrate content was determined by salicylic acid method (32).

### 2.4 Statistic analysis

The measurements of growth parameters were subjected to one-way analysis of variance (ANOVA) to test difference among means via SPSS software (SPSS Inc., Chicago, United States). Differences were assessed using the least significant difference calculations at a 5% confidence level in order to make treatment mean comparisons.

## 3 Results and discussion

### 3.1 The growth of *Chlorella vulgaris* cultivated under AC, HC, and MC

**Figure 1:**
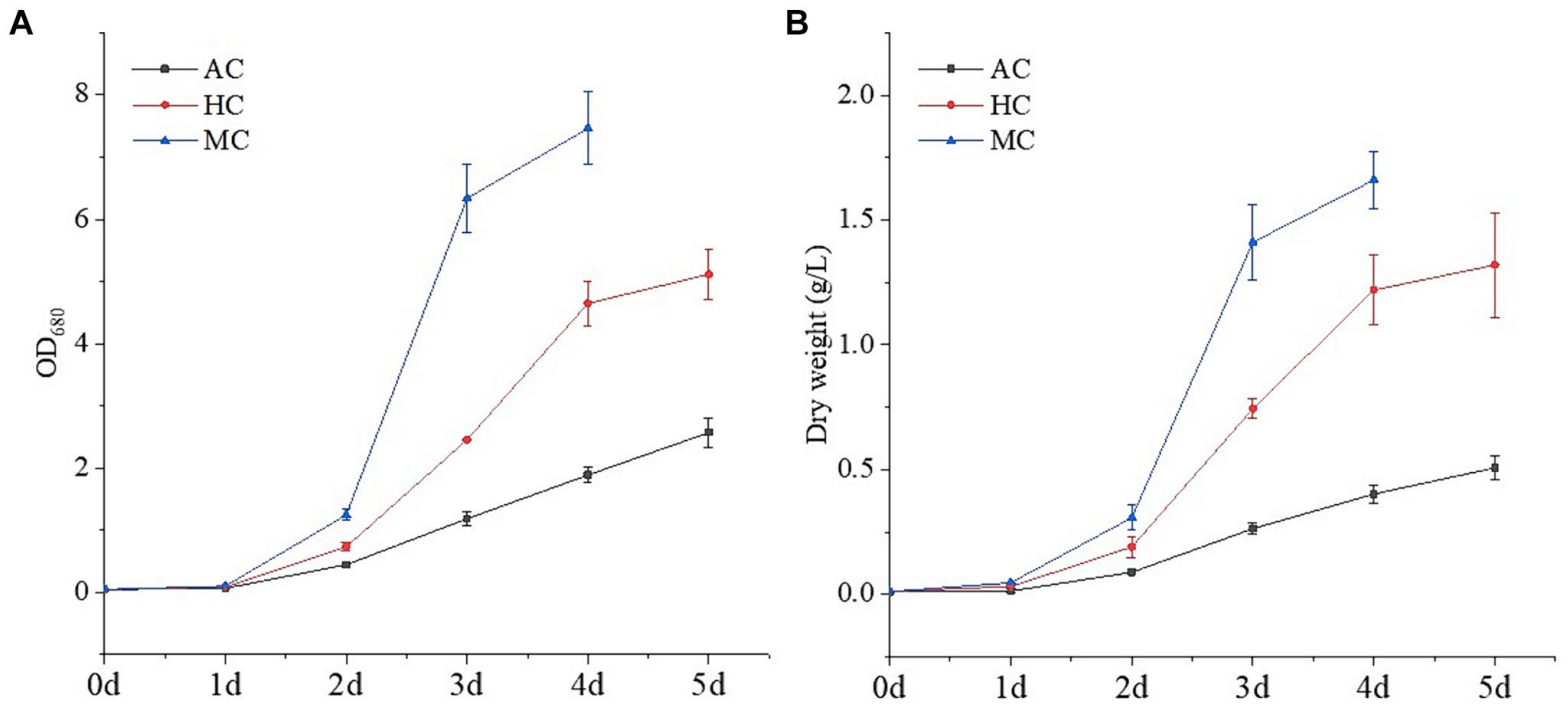
Growth curves of *C. vulgaris* under different cultural modes (autotrophic culture (AC), heterotrophic culture (HC) and mixotrophic culture (MC)): **(A)** OD_680_ and **(B)** dry weight. Different letters indicate significant difference (*p* < 0.05), according to one-way ANOVA.

Generally, according to carbon source and energy supply mode, microalgal cultivation methods can be divided into the following three types: AC, HC and MC. In this study, *C. vulgaris*, one of commercialized typical microalgal strains, was cultured under the three culture modes. The growth curves were shown in Figure 1. It could be seen that different culture modes had a great impact on the growth of *C. vulgaris*. For AC, the growth rate of *C. vulgaris* was relatively slow, and the dry weight was 0.51 g L^−1^ after incubation 5 day. *C. vulgaris* grown with CO_2_ as the unique carbon source, light provided all the energy required for biomass production. The efficiency of light harvesting, energy conversation and CO_2_ fixation limited the growth rate of *C. vulgaris* (33). Under HC and MC, *C. vulgaris* grew faster and the dry weights were 1.32 and 1.66 g L^−1^, which were 2.59 and 3.25 times that of AC, respectively. The growth characteristics of *C. vulgaris* under the three cultivation methods were consistent with the previous studies (33, 34). Many studies have shown that *C. vulgaris* can use glucose, glycerol, and acetate as carbon sources, and glucose is the most commonly used organic carbon source in microalgal culture, which produces more energy per mole compared to other carbon substrates (33). Therefore, the growth rates of *C. vulgaris* under MC and HC were significantly higher than that under AC. Compared with HC, the growth of *C. vulgaris* under MC could utilize organic carbon and inorganic carbon at the same time, which resulted in higher growth rate and biomass concentration. Wang et al. (35) reported that the growth of *C. vulgaris* were different under autotrophic, heterotrophic and mixotrophic culture. Heterotrophic and mixotrophic culture could significantly increase the biomass and specific growth rate of algal cells, which was more than 2 times that of autotrophic culture.

### 3.2 The composition of supernatant of *Chlorella vulgaris* cultivated under AC, HC, and MC

There were significant differences in the growth characteristics of microalgae and the nutrient utilization rate of the culture medium under the three culture methods, which resulted in the different content of residual nutrients in the culture medium. At the same time, the extracellular metabolites in the process of microalgal cultivation are different under different culture modes (33, 34, 36). Therefore, the obtained supernatant after microalgae harvest may produce different effects when it was used as fertilizer for crop planting.

As shown in Table 2, the nutrient contents of the supernatant were different under different culture modes. For macroelement, the concentrations of TN, TP, TK under AC were higher than that of under HC and MC. The concentration of TN under AC was 427.58 mg L^−1^, which were 2.79 and 3.66 times that of under HC and MC, respectively. On the contrary, the concentrations of TC under HC and MC were much higher than that of AC. However, the changes of Ca and Mg concentrations in different culture modes did not show a certain regularity. For trace elements, the concentrations of Fe and Mo under AC were higher than that of under HC and MC. The change of Cu concentration was opposite to that of Fe and Mo. As mentioned above, the *C. vulgaris* biomass of HC and MC was 3.25 and 2.59 times higher than that under AC, respectively. Thus, more biomass concentrations resulted in fewer remaining nutrient contents in the culture medium. The higher nutrient contents in the supernatant under AC than that under HC and MC might be caused by the lower biomass. However, TC concentrations of HC and MC were higher than those in AC, possibly due to the addition of 20 g L^−1^ glucose in the culture medium of HC and MC. Li et al. (37) also reported that the nitrogen utilization rate of *C. vulgaris* under autotrophic condition was significantly lower than that under heterotrophic and mixotrophic conditions, while the utilization rate of glucose under heterotrophic and mixotrophic conditions was basically the same.

**Table 2:** The nutrient contents of supernatant in AC, HC, and MC.

	HC	MC	AC
TC (mg/L)	7103.25b	8012.25a	272.90c
TN (mg/L)	153.22c	116.70b	427.58a
TP (mg/L)	2.02a	2.02a	2.20a
TK (mg/L)	0.97b	0.71c	1.56a
Mg (mg/L)	3.60a	3.10b	3.44ab
Ca (mg/L)	6.69a	5.24b	6.37a
Fe (mg/L)	0.39ab	0.31b	0.49a
Cu (μg/L)	9.14b	10.86a	7.74c
Mo (μg/L)	41.12b	43.98ab	47.10a

Different letters (a, b, c) indicate significant difference (*p* < 0.05), according to one-way ANOVA.

It has been proved that high amounts of metabolites could be secreted in the process of microalgal cultivation (38–40). The microalgae *C. vulgaris* is rich in chlorophyll, proteins, polysaccharides, vitamins, minerals, and essential amino acids (41). Amino acids are synthesized in microalgal cells and are also present in extracellular secretions. Granum et al. (42) investigated intracellular and extracellular production of amino acids by the marine diatom *Skeletonema costatum*. The results showed the composition of the extracellular free amino acids changed significantly from exponential to stationary growth phase. The proportions of acidic and small amino acids decreased, while large hydrophobic amino acids increased in accordance with intracellular changes. Perera et al. (43) investigated the effect of inorganic nitrogen sources on the extracellular metabolites of two microalgal strains *Tetradesmus obliquus* IS2 and *Coelastrella* sp. IS3. The composition of amino acids such as glutamate, aspartate, GABA and alanine varied with the ratios of nitrate and ammonium in the medium. Amino acids have many functions in agricultural cultivation, such as improving yield and yield components, improving nutrient assimilation and stress resistance, improving yield components and quality characteristics (44). Qu et al. (39) found that extracellular metabolites of heterotrophic *Auxenochlorella protothecoides* have obvious biostimulating effects on higher plant growth, and the content of amino acids in extracellular metabolites could reach to 0.102%. Table 3 showed the amino acid contents of supernatant in AC, HC and MC. It can be seen that the cultural conditions significantly affected the composition of amino acids in the supernatant. All the treatments contained 18 kinds of amino acids, including essential and non-essential amino acids. The essential amino acids accounted for 43.5, 57.0, and 29.3% of the total amino acids in AC, HC, and MC, respectively. HC had the highest the essential amino acids account rate compared with AC, the contents of amino acids in HC and MC were more similar. However, most of the contents of amino acids in MC treatment were much higher than that in HC and AC except for Cys and Met. These results indicated that light and carbon sources significantly affected the utilization of carbon and nitrogen by *C. vulgaris*, which resulted in the changes in the amino acid metabolism pathway. Han et al. (45) also reported the types and contents of metabolites of microalgae were greatly affected by different nutrient modes, which the biomass and lipid productivities of *C. vulgaris* with heterotrophic seed were 1.48 and 1.42 times higher than those with photoautotrophic seed. The nitrogen metabolism was linked with carbon metabolism in microalgae since they shared carbon supply and energy generated in the TCA cycle and in the mitochondrial electron transport chain (34, 46). Gao et al. (47) reported that the free amino acid contents were ranked from high to low as follows: mixotrophic group, heterotrophic group and autotrophic group. The mixotrophic group had the highest essential amino acid content between three modes.

**Table 3:** The amino acid content of supernatant in AC, HC, and MC.

ng/mL	HC	MC	AC
Asp	5.37b	10.44a	1.80c
Thr	6.66b	12.32a	1.46c
Ser	7.35b	11.39a	2.16c
Glu	15.77b	32.55a	11.51b
Gly	6.03b	33.44a	7.42b
Ala	35.44b	89.17a	30.40b
Cys	10.04a	4.33b	13.99a
Val	23.59b	37.18a	16.54c
Met	0.72a	0.40b	0.61a
Ile	5.72b	9.34a	0.24c
Leu	27.97a	40.03a	4.34b
Tyr	0.14b	1.39a	0.36b
Phe	93.02a	41.01b	35.60b
Lys	13.39b	27.08a	3.48c
His	1.21b	2.22a	0.33c
Arg	23.90b	43.04a	4.52c
Pro	0.50b	82.33a	0.46b
Asp	5.37b	10.44a	1.80c

Different letters (a, b, c) indicate significant difference (*p* < 0.05), according to one-way ANOVA.

Microalgal cells can secret phytohormones such as auxin, cytokinins, abscisic acid and ethylene, which play an important role in regulating cell growth, development and adapting to external environment (48). As shown in Tables 4, 5, all the treatments included indoles, cytokinins, abscisic acid, salicylic acid and gibberellic acid. Auxin is an essential regulator in various plant developmental processes. Its chemical essence is indoleacetic acid (IAA), which can promote growth at low concentration and inhibit growth and metabolism at high concentration (49). Indoles included IAA, IAAME, 13CA, IAId. The content of IAId was the highest among all types of phytohormones. Furthermore, the content of IAId was 399.27 ng/mL in MC, which was significantly higher than that in other treatments. AC had the lowest content of indoles since the slow growth rate and low biomass. Unlike the indoles content, the content of cytokinins in HC treatment was significantly higher than that in MC which included Zeatin, CZ, TZ, ZR, CZR, TZR, DZ, DZR, IP and IPR. Abscisic aicd (ABA) is a typical plant hormone. It is involved in abiotic stress response in plants, and plays an important role in regulating water balance and osmotic stress tolerance (50). The content of ABA showed no significant difference in AC, HC and MC ranged from 0.03 ng/mL to 0.06 ng/mL. Salicylic acid including SA and MESA in MC was higher than that in HC and AC although there was no significant difference. Moreover, SA was the main component in the supernatant varied from 13.20 ng/mL to 25.07 ng/mL under different cultural conditions. The most prominent role of gibberellin (GA) is to stimulate the elongation of cells and promote cell division, which can promote cell expansion (21). Moreover, the content of gibberellin (GA4) in MC was significantly higher than that in AC and HC. These phytohormones in the supernatant *C. vulgaris* might promote the growth of lettuce.

**Table 4:** The phytohormones content of supernatant in AC, HC, and MC.

ng/mL	HC	MC	AC
IAA	0.20a	0.42a	0.14a
IAAME	1.80a	2.22a	1.97a
13CA	4.73a	8.38a	4.011a
IAId	75.61b	399.27a	30.20b
Zeatin	0.18a	0.06b	0.04b
CZ	0.14a	0.06b	0.02b
TZ	0.03a	0.02a	0.03a
ZR	0.12a	0.05b	0.03b
CZR	0.09a	0.04b	0.02b
TZR	0.03a	0.01b	0.01b
DZ	0.04a	0.02b	0.02b
DZR	0.04a	0.02ab	0.01b
IP	5.55a	2.06b	0.18c
IPR	2.17a	0.53b	0.39b
ABA	0.04b	0.06a	0.03b
SA	14.95a	24.72a	12.82a
MESA	0.20a	0.35a	0.38a
GA4	1.14b	3.61a	0.86b

Different letters (a, b, c) indicate significant difference (*p* < 0.05), according to one-way ANOVA.

**Table 5:** Different categories of phytohormones in AC, HC, and MC.

ng/mL	HC	MC	AC
Indoles	82.33b	410.29a	36.32b
Cytokinins	8.38a	2.86b	0.73b
Abscisic acid	0.04b	0.06a	0.03b
Salicylic acid	15.15a	25.07a	13.20a
Gibberellic acid	1.14b	3.61a	0.86b

Indoles (IAA, IAAME, 13CA, IAId), Cytokinins (Zeatin, CZ, TZ, ZR, CZR, TZR, DZ, DZR, IP, IPR), Abscisic acid (ABA), Salicylic acid (SA, MESA), Gibberellic acid (GA4). Different letters (a, b, c) indicate significant difference (*p* < 0.05), according to one-way ANOVA.

### 3.3 The effect of supernatant on the growth of lettuce

**Figure 2:**
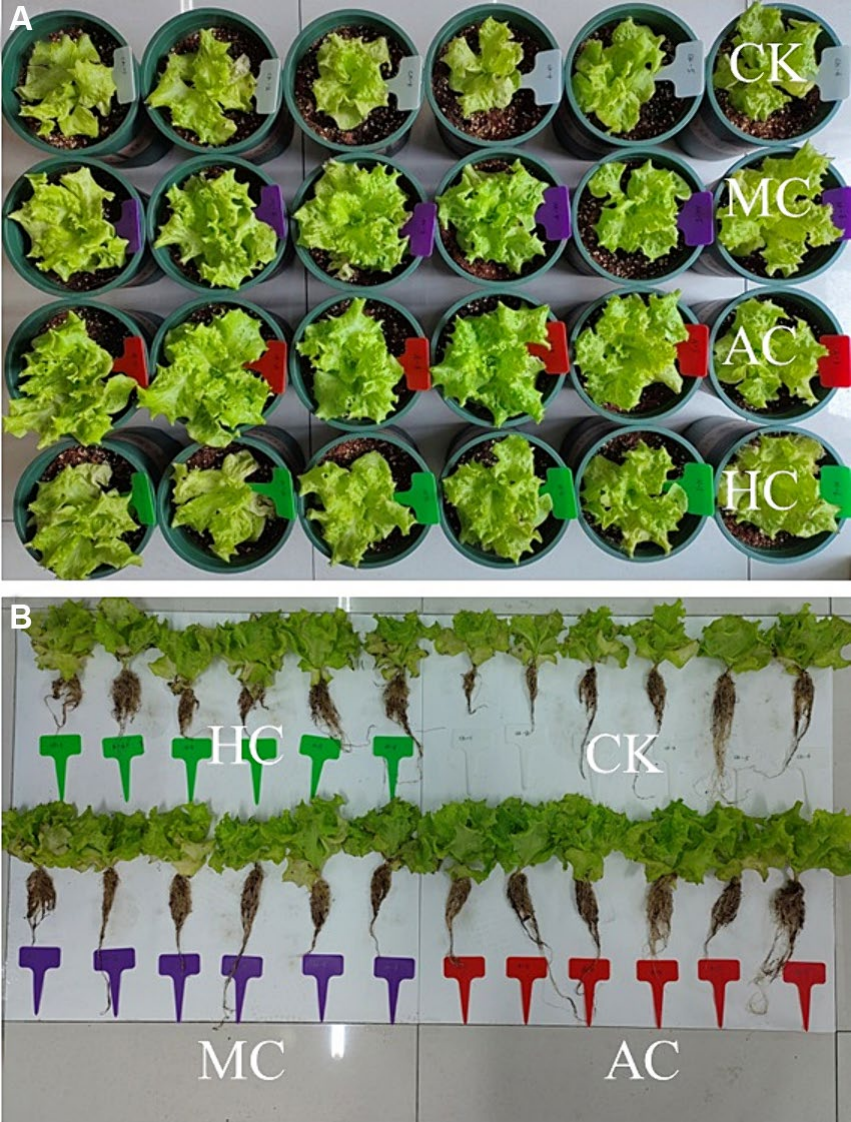
Effects of supernatant obtained from different cultivation modes (autotrophic culture (AC), heterotrophic culture (HC) and mixotrophic culture (MC)) on the development of shoot **(A)** and root **(B)** of lettuce. Different letters indicate significant difference (*p* < 0.05), according to one-way ANOVA.

In order to investigate the possibility of supernatant as fertilizer, the lettuce planting experiment was carried out with the supernatant obtained under three culture modes, and with deionized water as the control. As shown in Figure 2, the groups treated with supernatant exhibited a growth-promoting effect compared with the control group. The supernatant obtained from different culture modes also had different effects on the growth of lettuce.

**Figure 3:**
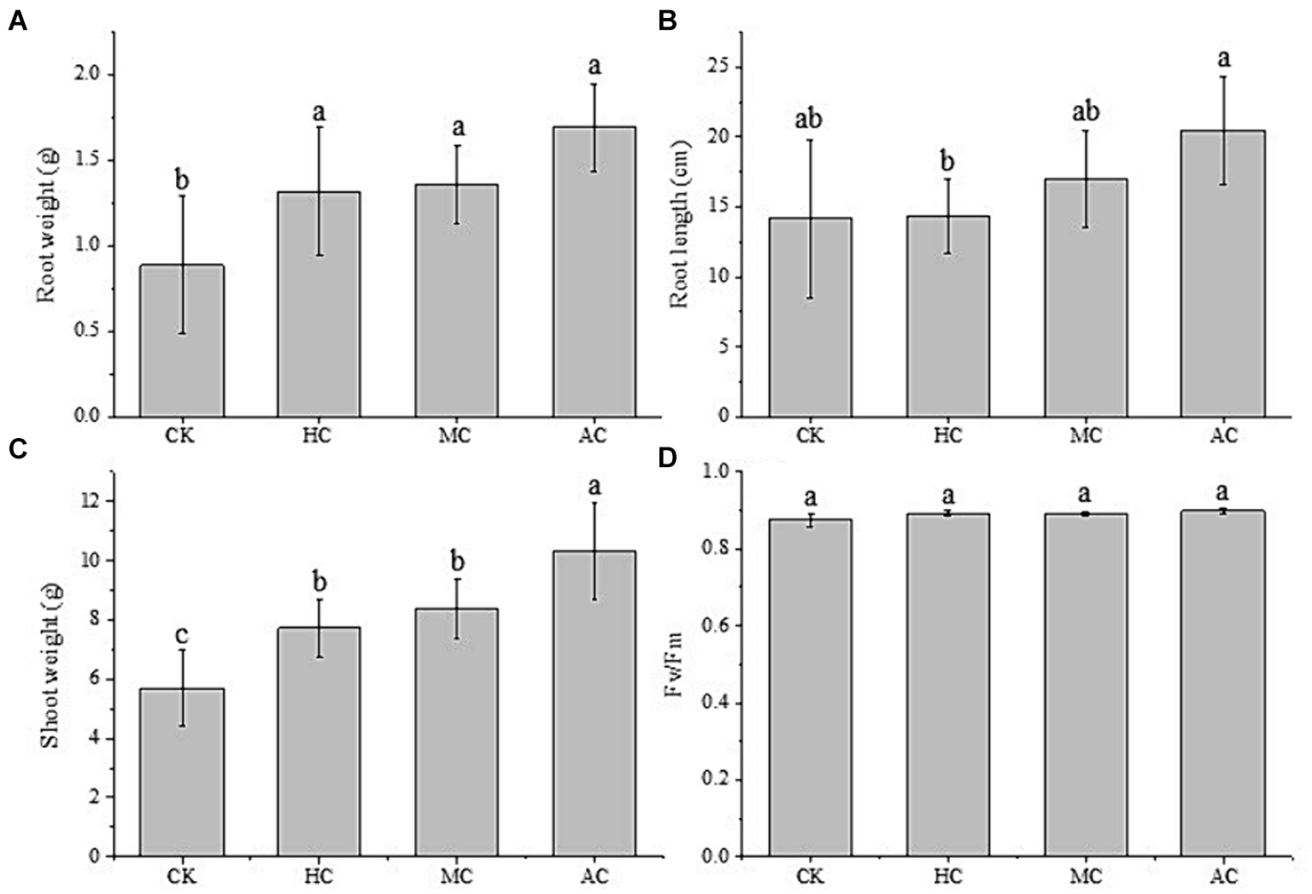
Effects of supernatant obtained from different cultivation modes (autotrophic culture (AC), heterotrophic culture (HC) and mixotrophic culture (MC)) on the root weight **(A)**, root length **(B)**, shoot length **(C)**, and *F*_v_/*F*_m_ **(D)** of lettuce. Different letters indicate significant difference (*p* < 0.05), according to one-way ANOVA.

The fresh root and shoot weight, and the root length of lettuce were measured and shown in Figure 3. It can be seen that the fresh root weights of lettuce treated with the supernatant were higher compared with the control group. The lettuce treated with the supernatant of AC had the highest root weight, and there was no significant difference among the three different culture modes. However, they were significantly higher than the control group, with an increase of 48.5, 53.4, and 91.0%, respectively (Figure 3A). At the same time, the application of supernatant also promoted the root length. As shown in Figure 3B, it can be seen that the root length of lettuce treated with the supernatant of AC was significantly higher than that of other treatments. The changes of the fresh shoot weight of lettuce were consistent with that of root. The three supernatant treatment groups were significantly higher than the control group. Compared with the control group, the three supernatant treatment groups increased by 35.8, 47.0, and 81.0%, respectively. The fresh shoot weight of lettuce treated with the supernatant of AC reached to 10.31 g, and was significantly higher than that of lettuce treated with the supernatant of HC and MC.

Chlorophyll fluorescence parameters can reflect the changes of light utilization of plants under environmental stress (51). The *F*_v_/*F*_m_ values of lettuce were measured and shown in Figure 3D, and it was found that *F*_v_/*F*_m_ values of all treatment groups were between 0.88–0.90. There was no significant difference between all the treatment groups, which indicated the nutrients in each treatment group, including the control group, did not affect the normal photosynthesis of lettuce.

**Figure 4:**
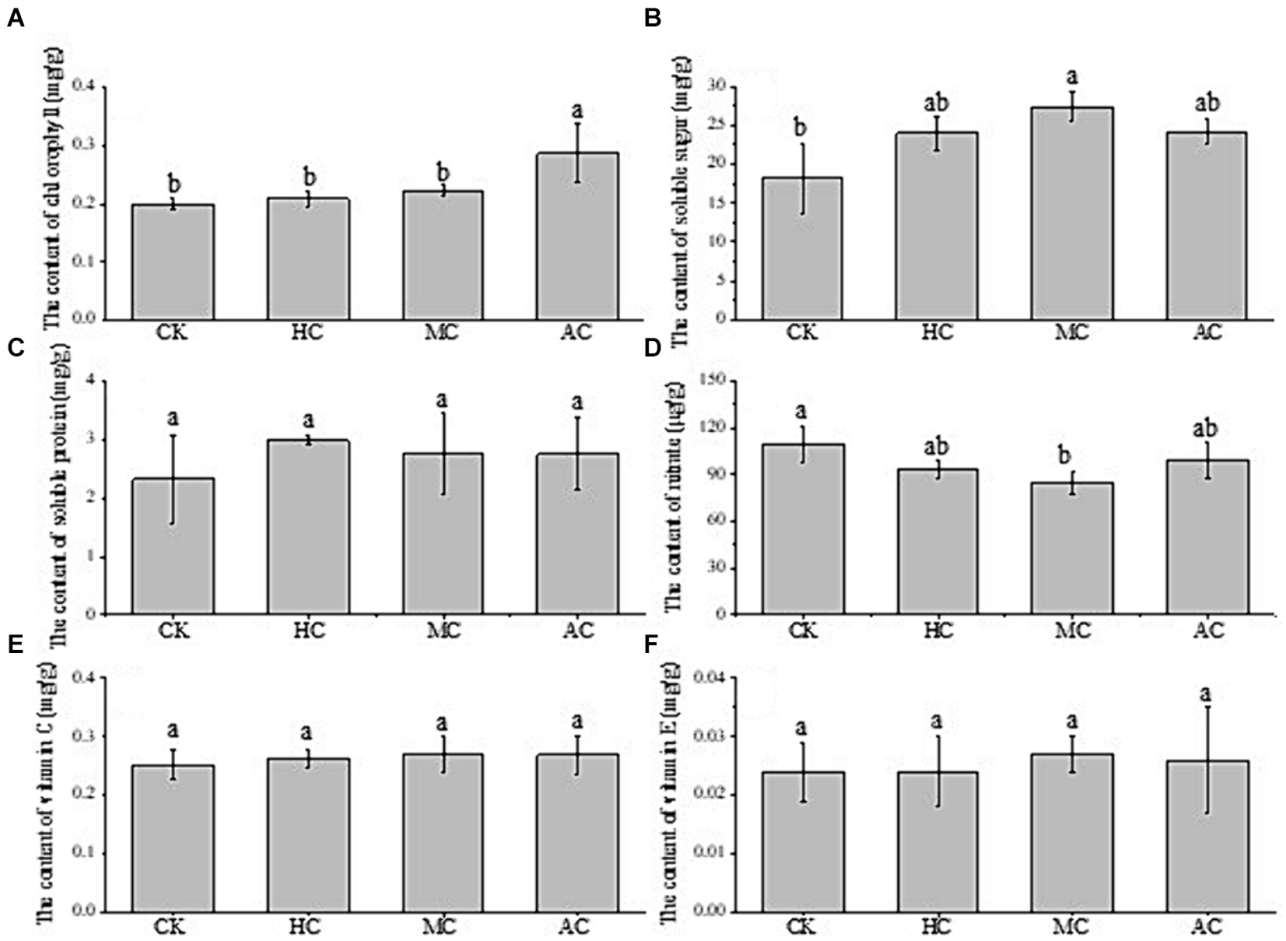
Effect of supernatant obtained from different cultivation modes (autotrophic culture (AC), heterotrophic culture (HC) and mixotrophic culture (MC)) on the contents of chlorophyll **(A)**, soluble sugar **(B)**, soluble protein **(C)**, nitrate **(D)**, vitamin C **(E)** and vitamin E **(F)** of lettuce. Different letters indicate significant difference (*p* < 0.05), according to one-way ANOVA.

Figure 4 showed the effects of different treatments on the nutrient contents of lettuce. It can be seen that the chlorophyll content of AC was significantly higher than that of the other three groups, and there was no significant difference between the other three groups (Figure 4A). The content of soluble sugar in MC group was the highest and significantly higher than that in the control group, but there was no significant difference compared with AC and HC groups (Figure 4B). The supernatant of *C. vulgaris* increased the protein content of lettuce, but there was no significant difference (Figure 4C). As shown in Figure 4D, the nitrate content of lettuce in the control group was highest among all the treatment groups, and significantly higher than that of MC group which indicated that supernatant might have the positive effects on reducing nitrate accumulation in lettuce. Excessive nitrate can be reduced to nitrite, which can be further transformed into a strong carcinogen nitrosamine, causing cancer. A large number of studies have shown that 60%–80% of nitrate consumed by human body coming from vegetables (52). Lettuce is easy to accumulate nitrate, so the nitrate content of lettuce is a very important index to evaluate the quality of lettuce. Vitamin C (VC) and vitamin E (VE) have antioxidant effects and are indispensable substances for human body (53). There was no significant difference in the contents of VC and VE among all the treatments, indicating that the supernatant could not improve the contents of VC and VE in lettuce (Figures 4E, F).

### 3.4 Pearson’s correlations between lettuce growth parameters and supernatant nutrients

Table 6 showed the relationship between phytohormones content of supernatant and the growth parameters of lettuce under different treatments. It can be seen that the chlorophyll content and shoot weight of lettuce was significantly positive with the most nutrients and phytohormones except for Ca, Cu, Mo and cytokinins. Mutale-Joan et al. (54) evaluated the effects on nutrient uptake of extracts obtained from microalgae on tomato seedlings. The results showed that root N concentration was more closely associated to shoot dry weight and chlorophyll content in leaves, while P and K levels in roots were closely associated with enhanced root length. Compared to control treatment, the nutrient content of supernatant improved the lettuce growth, and this was the reason why the shoot weight of lettuce was significantly higher treated with supernatant from different cultures than that in control treatment. Haroun and Hussein (55) reported that treating seed priming of *Lupinus termis* with culture filtrates of two blue-green algae led to an increase in chlorophylls in leaves, consequently increasing the photosynthetic activity, content of carbohydrates and nitrogenous compounds in the shoot.

**Table 6:** Pearson’s correlations between lettuce growth parameters and supernatant nutrients.

	Chl	Sugar	Protein	NO_3_^−^-N	VC	VE	Shoot weight	Root weight	Root length	*F*_v_/*F*_m_
Na	0.67*	0.34	−0.08	−0.24	−0.01	0.03	0.64*	0.48	0.73**	0.60*
Mg	0.64*	0.32	0.04	−0.16	0.10	−0.12	0.79**	0.22	0.19	0.55
N	0.63*	0.29	0.03	−0.35	0.04	0.04	0.73**	0.56	0.44	0.42
P	0.72**	0.44	0.37	−0.52	0.33	−0.06	0.74**	0.13	0.25	0.58*
K	0.71**	0.27	0.19	−0.19	0.20	0.08	0.79**	0.37	0.37	0.72**
Ca	0.39	0.10	0.09	−0.16	0.03	0.17	0.51	0.07	0.13	0.71*
Fe	0.69*	0.16	0.05	−0.27	0.08	−0.13	0.82**	0.33	0.24	0.51
Cu	0.11	0.62*	0.09	−0.58*	0.06	0.28	0.20	0.06	0.16	0.18
Mo	0.49	0.32	0.04	−0.46	−0.03	−0.07	0.64*	0.49	0.32	0.24
Indoles	0.87**	0.22	0.15	0.08	0.23	0.30	0.73**	0.64*	0.46	0.51
Cytokinins	−0.05	0.16	0.30	−0.14	−0.02	−0.08	0.15	−0.30	−0.32	0.33
Abscisicacid	0.70*	0.44	0.11	−0.25	0.15	0.16	0.80**	0.62*	0.42	0.58*
Salicylicacid	0.76**	0.43	0.12	−0.13	0.18	0.36	0.64*	0.65*	0.46	0.48
Gibberellic	0.62*	0.25	0.15	−0.08	0.05	−0.03	0.74**	0.49	0.37	0.59*

** significant at *p* < 0.01 and * significant at *p* < 0.05.

The content of NO_3_^−^N was negatively with almost all the factors, moreover, it was significantly negative with Cu content of supernatant. Differently, sugar content showed a significant positive relationship with Cu. It was noteworthy that the shoot weight was significantly influenced by the supernatant nutrients and phytohormones. Compared with control treatment, whether autotrophic or heterotrophic treatment had different amounts of biostimulants which could enhance plant growth and reduce biotic and abiotic stresses (22). It was found that the root weight was significantly positive with abscisic acid and indoles, and the root length was positive with abscisic acid although it was not significant. It has also been reported that high levels of abscisic acid was found to promote root elongation and growth through suppressing ethylene synthesis, which in turn reduces IAA transport and biosynthesis in the root tip (56).

## 4 Conclusion

In this study, *C. vulgaris* was cultured under three common microalgal culture methods, and the composition of the supernatant of *C. vulgaris* culture were analyzed and compared. The supernatant was used in the pot experiment of lettuce, ant the results showed that the supernatant could promote the growth of lettuce. It provides a solution for the utilization of supernatant after microalgae harvesting.

## Data Availability

The raw data supporting the conclusions of this article will be made available by the authors, without undue reservation.

## References

[1] Cheah WY Show PL Chang J-S Ling TC Juan JC. Biosequestration of atmospheric CO_2_ and flue gas-containing CO_2_ by microalgae. Bioresour Technol. (2015) 184:190–201. doi: 10.1016/j.biortech.2014.11.026, PMID: 25497054

[2] Venkata SG Rajvanshi M Navish KB Govindachary S Prasad V Dasgupta S. Carbon streaming in microalgae: extraction and analysis methods for high value compounds. Bioresour Technol. (2017) 244:1304–16. doi: 10.1016/j.biortech.2017.07.024, PMID: 28803061

[3] Lu Z Loftus S Sha J Wang W Park MS Zhang X. Water reuse for sustainable microalgae cultivation: current knowledge and future directions. Resour Conserv Recycl. (2020) 161:104975. doi: 10.1016/j.resconrec.2020.104975

[4] Yang J Xu M Zhang XZ Hu Q Sommerfeld M Chen YS. Life-cycle analysis on biodiesel production from microalgae: water footprint and nutrients balance. Bioresour Technol. (2011) 102:159–65. doi: 10.1016/j.biortech.2010.07.017, PMID: 20675125

[5] Depraetere O Pierre G Noppe W Vandamme D Foubert I Michaud P. Influence of culture medium recycling on the performance of arthrospira platensis cultures. Algal Res. (2015) 10:48–54. doi: 10.1016/j.algal.2015.04.014

[6] Lu Z Sha J Wang W Li Y Wang G Chen Y. Identification of auto-inhibitors in the reused culture media of the Chlorophyta Scenedesmus acuminatus. Algal Res. (2019) 44:101665. doi: 10.1016/j.algal.2019.101665

[7] Rodolfi L Zittelli GC Barsanti L Rosati G Tredici MR. Growth medium recycling in Nannochloropsis sp. mass cultivation. Biomol Eng. (2003) 20:243–8. doi: 10.1016/S1389-0344(03)00063-7, PMID: 12919804

[8] Farooq W Suh WI Park MS Yang JW. Water use and its recycling in microalgae cultivation for biofuel application. Bioresour Technol. (2015) 184:73–81. doi: 10.1016/j.biortech.2014.10.140, PMID: 25465788

[9] Fret J Roef L Blust R Diels L Tavernier S Vyverman W. Reuse of rejuvenated media during laboratory and pilot scale cultivation of Nannochloropsis sp. Algal Res. (2017) 27:265–73. doi: 10.1016/j.algal.2017.09.018

[10] Gonzalez-Lopez CV Ceron-Garcia MC Fernandez-Sevilla JM Gonzalez-Cespedes AM Camacho-Rodriguez J Molina-Grima E. Medium recycling for nannochloropsis gaditana cultures for aquaculture. Bioresour Technol. (2013) 129:430–8. doi: 10.1016/j.biortech.2012.11.061, PMID: 23262021

[11] Hadj-Romdhane F Zheng X Jaouen P Pruvost J Grizeau D Croue JP. The culture of Chlorella vulgaris in a recycled supernatant: effects on biomass production and medium quality. Bioresour Technol. (2013) 132:285–92. doi: 10.1016/j.biortech.2013.01.025 23411460

[12] Zhang XZ Lu ZY Wang YF Wensel P Sommerfeld M Hu Q. Recycling Nannochloropsis oceanica culture media and growth inhibitors characterization. Algal Res. (2016) 20:282–90. doi: 10.1016/j.algal.2016.09.001

[13] Fret J Roef L Diels L Tavernier S Vyverman W Michiels M. Implementation of flocculation and sand filtration in medium recirculation in a closed microalgae production system. Algal Res. (2016) 13:116–25. doi: 10.1016/j.algal.2015.11.016

[14] Morocho-Jácome AL Mascioli GF Sato S Carvalho JCMD. Continuous cultivation of arthrospira platensis using exhausted medium treated with granular activated carbon. J Hydrol. (2015) 522:467–74. doi: 10.1016/j.jhydrol.2015.01.001

[15] Morocho-Jácome AL Mascioli GF Sato S de Carvalho JCM. Evaluation of physicochemical treatment conditions for the reuse of a spent growth medium in arthrospira platensis cultivation. Algal Res. (2016) 13:159–66. doi: 10.1016/j.algal.2015.11.022

[16] Monte J Sa M Parreira C Galante J Serra AR Galinha CF. Recycling of Dunaliella salina cultivation medium by integrated membrane filtration and advanced oxidation. Algal Res. (2019) 39:101460. doi: 10.1016/j.algal.2019.101460

[17] Wang WX Sha J Lu ZY Shao SL Sun PZ Hu Q. Implementation of UV-based advanced oxidation processes in algal medium recycling. Sci Total Environ. (2018) 634:243–50. doi: 10.1016/j.scitotenv.2018.03.342, PMID: 29627547

[18] Behera B Supraja KV Paramasivan B. Integrated microalgal biorefinery for the production and application of biostimulants in circular bioeconomy. Bioresour Technol. (2021) 339:125588. doi: 10.1016/j.biortech.2021.125588 34298244

[19] Coppens J Grunert O Van Den Hende S Vanhoutte I Boon N Haesaert G. The use of microalgae as a high-value organic slow-release fertilizer results in tomatoes with increased carotenoid and sugar levels. J Appl Phycol. (2016) 28:2367–77. doi: 10.1007/s10811-015-0775-2

[20] Gitau MM Farkas A Balla B Ördög V Futó Z Maróti G. Strain-specific biostimulant effects of Chlorella and Chlamydomonas green microalgae on Medicago truncatula. Plan Theory. (2021) 10:1060. doi: 10.3390/plants10061060, PMID: 34070559 PMC8227499

[21] Kapoore RV Wood EE Llewellyn CA. Algae biostimulants: a critical look at microalgal biostimulants for sustainable agricultural practices. Biotechnol Adv. (2021) 49:107754. doi: 10.1016/j.biotechadv.2021.107754, PMID: 33892124

[22] Lu Y Xu J. Phytohormones in microalgae: a new opportunity for microalgal biotechnology? Trends Plant Sci. (2015) 20:273–82. doi: 10.1016/j.tplants.2015.01.006, PMID: 25697753

[23] Mieczyslaw G Romanowska-Duda Z. Improvements in germination, growth, and metabolic activity of corn seedlings by grain conditioning and root application with Cyanobacteria and microalgae. Pol J Environ Stud. (2014) 23:1147–53.

[24] Puglisi I Barone V Fragalà F Stevanato P Baglieri A Vitale A. Effect of microalgal extracts from Chlorella vulgaris and Scenedesmus quadricauda on germination of Beta vulgaris seeds. Plan Theory. (2020) 9:675. doi: 10.3390/plants9060675, PMID: 32466497 PMC7355607

[25] Ronga D Biazzi E Parati K Carminati D Carminati E Tava A. Microalgal biostimulants and biofertilisers in crop productions. Agronomy. (2019) 9:192. doi: 10.3390/agronomy9040192

[26] Zelazny LW He L Vanwormhoudt AM. Methods of soil analysis, part 3: Chemical methods. USA: Madison (1996).

[27] Mittal M Kumar K Anghore D Rawal RK. ICP-MS: analytical method for identification and detection of elemental impurities. Curr Drug Discov Technol. (2017) 14:106–20. doi: 10.2174/1570163813666161221141402, PMID: 28003007

[28] Aron S Thorsten D. Low volume quantification of dissolved organic carbon and dissolved nitrogen. Limnol Oceanogr Methods. (2012) 10:347–52. doi: 10.4319/lom.2012.10.347

[29] Watanabe K Badr ES Pan X Achterberg EP. Conversion efficiency of the high-temperature combustion technique for dissolved organic carbon and total dissolved nitrogen analysis. Int J Environ An Ch. (2007) 87:387–99. doi: 10.1080/03067310701237023

[30] Babu SVS Shareef MM Shetty APK Shetty KT. HPLC method for amino acids profile in biological fluids and inborn metabolic disorders of aminoacidopathies. Indian J Clin Biochem. (2002) 17:7–26. doi: 10.1007/bf02867967, PMID: 23105346 PMC3454125

[31] Wu Y Shen Y. Dormancy in Tilia miqueliana is attributable to permeability barriers and mechanical constraints in the endosperm and seed coat. Braz J Bot. (2021) 44:725–40. doi: 10.1007/s40415-021-00749-1

[32] Zhao L Wang Y. Nitrate assay for plant tissues. Bio-Protocol. (2017) 7:e2029. doi: 10.21769/BioProtoc.2029, PMID: 34458432 PMC8376535

[33] Velea S. Oancea F. Fischer F., (2017). Heterotrophic and mixotrophic microalgae cultivation. Woodhead publishing, 45–65

[34] Perez-Garcia O Escalante FME De-Bashan LE Bashan Y. Heterotrophic cultures of microalgae: metabolism and potential products. Water Res. (2011) 45:11–36. doi: 10.1016/j.watres.2010.08.037 20970155

[35] Wang H Guo S Zheng B Li C. Growth and biochemical components of Chlorella vulgaris under autotrophic, heterotrophic and mixotrophic cultivations. J South China Univ Technol. (2004) 32:47–50. doi: 10.1161/01.ATV.0000116865.98067.31

[36] Zheng YB Chi ZY Lucker B Chen SL. Two-stage heterotrophic and phototrophic culture strategy for algal biomass and lipid production. Bioresour Technol. (2012) 103:484–8. doi: 10.1016/j.biortech.2011.09.122, PMID: 22023968

[37] Li C Yang H Li Y Wang W. Effect of culture models on metabolism and protein components of microalgae Chlorella vulgaris (in Chinese). J Food Sci Biotechnol. (2014) 33:56–62. doi: 10.3969/j.issn.1673-1689.2014.01.009

[38] Ballesteros-Torres J Samaniego-Moreno L Gomez-Flores R Tamez-Guerra R Rodriguez-Padilla C Tamez-Guerra P. Amino acids and acylcarnitine production by Chlorella vulgaris and Chlorella sorokiniana microalgae from wastewater culture. PeerJ. (2019) 7:e7977. doi: 10.7717/peerj.7977 31824754 PMC6896938

[39] Qu Y Chen X Ma B Zhu H Zheng X Yu J. Extracellular metabolites of heterotrophic auxenochlorella protothecoides: a new source of bio-stimulants for higher plants. Mar Drugs. (2022) 20:569. doi: 10.3390/md20090569 36135758 PMC9505405

[40] Stirk W van Staden J. Bioprospecting for bioactive compounds in microalgae: antimicrobial compounds. Biotechnol Adv. (2022) 59:107977. doi: 10.1016/j.biotechadv.2022.107977, PMID: 35580750

[41] De Morais MG Vaz BDS De Morais EG Costa JAV. Biologically active metabolites synthesized by microalgae. Biomed Res Int. (2015) 2015:835761:1–15. doi: 10.1155/2015/835761, PMID: 26339647 PMC4538420

[42] Granum E Kirkvold S Myklestad SM. Cellular and extracellular production of carbohydrates and amino acids by the marine diatom Skeletonema costatum: diel variations and effects of N depletion. Mar Ecol Prog Ser. (2002) 242:83–94. doi: 10.3354/meps242083

[43] Perera IA Abinandan S Subashchandrabose SR Venkateswarlu K Naidu R Megharaj M. Combined inorganic nitrogen sources influence the release of extracellular compounds that drive mutualistic interactions in microalgal-bacterial co-cultures. J Appl Phycol. (2022) 34:1311–22. doi: 10.1007/s10811-022-02711-4

[44] Sun W Shahrajabian MH Kuang Y Wang N. Amino acids biostimulants and protein hydrolysates in agricultural sciences. Plan Theory. (2024) 13:210. doi: 10.3390/plants13020210, PMID: 38256763 PMC10819947

[45] Han F Huang J Li Y Wang W Wang J Fan J. Enhancement of microalgal biomass and lipid productivities by a model of photoautotrophic culture with heterotrophic cells as seed. Bioresour Technol. (2012) 118:431–7. doi: 10.1016/j.biortech.2012.05.066, PMID: 22717560

[46] Fernandez E Galvan A. Inorganic nitrogen assimilation in Chlamydomonas. J Exp Bot. (2007) 58:2279–87. doi: 10.1093/jxb/erm106, PMID: 17578869

[47] Gao J Zhang W Dou Y Jiang Z Jia X Shao P. Effects of culture mode on fatty acid and amino acids in Euglena gracilis (in Chinese). Acta Hydrobiologica Sinica. (2020) 3:631–7. doi: 10.7541/2020.077

[48] Wang C Qi M Guo J Zhou C Yan X Ruan R. The active Phytohormone in microalgae: the characteristics, efficient detection, and their adversity resistance applications. Molecules. (2022) 27:46. doi: 10.3390/molecules27010046, PMID: 35011277 PMC8746318

[49] Hashtroudi MS Ghassempour A Riahi H Shariatmadari Z Khanjir M. Endogenous auxins in plant growth-promoting Cyanobacteria—Anabaena vaginicola and Nostoc calcicola. J Appl Phycol. (2013) 25:379–86. doi: 10.1007/s10811-012-9872-7

[50] Dar NA Amin I Wani W Wani SA Shikari AB Wani SH. Abscisic acid: a key regulator of abiotic stress tolerance in plants. Plant Gene. (2017) 11:106–11. doi: 10.1016/j.plgene.2017.07.003

[51] Bhagooli R Mattan-Moorgawa S Kaullysing D Louis Y Gopeechund A Ramah S. Chlorophyll fluorescence-a tool to assess photosynthetic performance and stress photophysiology in symbiotic marine invertebrates and seaplants. Mar Pollut Bull. (2021) 165:112059. doi: 10.1016/j.marpolbul.2021.112059 33677415

[52] Zhang J Li H Deng J Wang L. Assessing impacts of nitrogen management on nitrous oxide emissions and nitrate leaching from greenhouse vegetable systems using a biogeochemical model. Geoderma. (2021) 382:114701. doi: 10.1016/j.geoderma.2020.114701

[53] Pala Ş Atilgan R Kuloğlu T Kara M Başpinar M Can B. Protective effects of vitamin C and vitamin E against hysterosalpingography-induced epithelial degeneration and proliferation in rat endometrium. Drug Des Devel Ther. (2016) 10:4079–89. doi: 10.2147/DDDT.S117207, PMID: 28008231 PMC5170617

[54] Mutale-joan C Redouane B Najib E Yassine K Lyamlouli K Laila S. Screening of microalgae liquid extracts for their biostimulant properties on plant growth, nutrient uptake and metabolite profile of Solanum lycopersicum L. Sci Rep. (2020) 10:2820. doi: 10.1038/s41598-020-59840-4, PMID: 32071360 PMC7028939

[55] Haroun SA Hussein MH. The promotive effect of algal biofertilizers on growth, protein pattern and some metabolic activities of Lupius termis plants grown in siliceous soil. Asian J Plant Sci. (2003) 2:944–51. doi: 10.3923/ajps.2003.944.951

[56] McAdam SAM Brodribb TJ Ross JJ. Shoot-derived abscisic acid promotes root growth. Plant, Cell and Environ. (2016) 39:652–9. doi: 10.1111/pce.12669 26514625

